# The Impact of Contact Tracing in Clustered Populations

**DOI:** 10.1371/journal.pcbi.1000721

**Published:** 2010-03-26

**Authors:** Thomas House, Matt J. Keeling

**Affiliations:** Warwick Mathematics Institute and Department of Biological Sciences, University of Warwick, Coventry, United Kingdom; University of Texas at Austin, United States of America

## Abstract

The tracing of potentially infectious contacts has become an important part of the control strategy for many infectious diseases, from early cases of novel infections to endemic sexually transmitted infections. Here, we make use of mathematical models to consider the case of partner notification for sexually transmitted infection, however these models are sufficiently simple to allow more general conclusions to be drawn. We show that, when contact network structure is considered in addition to contact tracing, standard “mass action” models are generally inadequate. To consider the impact of mutual contacts (specifically clustering) we develop an improvement to existing pairwise network models, which we use to demonstrate that *ceteris paribus*, clustering improves the efficacy of contact tracing for a large region of parameter space. This result is sometimes reversed, however, for the case of highly effective contact tracing. We also develop stochastic simulations for comparison, using simple re-wiring methods that allow the generation of appropriate comparator networks. In this way we contribute to the general theory of network-based interventions against infectious disease.

## Author Summary

There are multiple ways to control infectious diseases—vaccination and drugs such as antibiotics or anti-virals form part of the pharmaceutical approach, however another route is to stop people infecting each other. This can be done either through general efforts to reduce epidemiologically relevant contacts, or through a more targeted attempt to trace the contacts of known cases who can then be isolated or treated. The impact of this kind of contact tracing is *a priori* likely to depend strongly on the network of contacts linking people together. In this paper, we develop new mathematical and computational techniques to model the impact of clustering: the probability that any two contacts of a given individual are also linked to each other in the network, creating triangles. Often, and for intuitively understandable reasons, the presence of clustering increases the efficacy of contact tracing, however we show that in the regime of highly effective contact tracing sometimes the opposite is true.

## Introduction

Modelling has become a central tool in understanding the epidemiology of infectious disease, and designing control strategies. One control method, contact tracing, has been considered in a large number of disease contexts. These include the 2003 SARS pandemic [1],[2], the 2001 UK FMD epidemic [3]–[6], contingency planning for deliberate release of smallpox [7],[8], and control of sexually transmitted infections [9]–[11]. A particular benefit of tracing is that it allows targeting of control, at the cost of effort spent on finding the individuals at risk.

Since contact tracing takes place as a process over the network of interactions between hosts, it is natural to consider network-based models of this process. Theoretical work has so far dealt with contact tracing as a branching process [12], through modifications to mean-field equations [13], pairwise approximations [14] and simulation [15]. This work means that the implications of heterogeneous numbers of contacts (and related network properties such as assortativity) for the efficacy of contact tracing are reasonably well understood.

For the case of clustering, due to the analytical challenge posed by the existence of short closed loops in the contact network, it has generally been more difficult to make similar progress. Existing theoretical work has therefore either been restricted to the ‘limiting case’ of clump structured populations, with all clustering due to completely connected cliques [16], or else simulation on exemplar networks [13],[14],[17].

In this work, we derive an improved triple closure for clustered pairwise models that removes two significant problems with existing closure regimes, and use this to make a systematic investigation of the impact of clustering on the efficacy of contact tracing, keeping other network and epidemiological parameters constant as appropriate. We find that, for many parameter choices, there are intuitive explanations, borne out by modelling, for the increased impact of contact tracing as clustering increases. This is not, however, a completely general result, meaning that the full implications of clustering for the efficacy of contact tracing are subtle and should be the subject of case by case investigation.

We perform our analysis within the *SIS* paradigm, meaning that while some of our terminology will be general to all infectious disease epidemiology, other statements will be geared towards the modelling of sexually transmitted infections where recovery/treatment does not confer lasting immunity.

## Methods

### Modelling contact tracing

**Figure 1:**
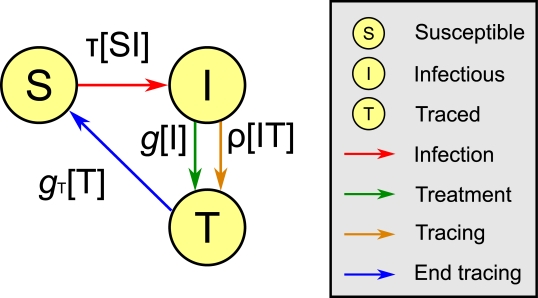
The compartments and processes for *SIS* dynamics with contact tracing. Processes are shown with coloured arrows, labelled according to the rate at which they happen.

The dynamics underpinning our model are shown schematically in Figure 1. Individuals are either susceptible (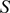), infectious (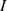) or traced (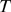) and move between these compartments due to four processes: infection; treatment; tracing; and stopping tracing. This paradigm is suitable for the consideration of sexually transmitted diseases, where infectious individuals can transmit infection to contacts, then seek treatment, which clears the pathogen and stops transmission but leaves the individual susceptible. It also involves the process of contact tracing, which we use as a general term that includes both partner notification and efforts by public-health workers to track down potentially infected individuals.

The four processes described so far separate into two categories: those that happen at an individual level, and contact processes. Seeking treatment and the cessation of tracing take place in the population at rates proportional to a number of individuals, and so fall into the former category. Using square brackets around a quantity to indicate its expected number in the population (so that quantities in square brackets are extensive expected numbers rather than intensive proportions) we take treatment to happen at a rate 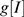, where 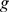 is the treatment rate constant, and the cessation of efforts to trace a treated individual's contacts to happen at rate 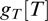, where 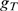 is the rate constant associated with the end of tracing.

Infection and contact tracing, on the other hand, are contact processes, and so take place at a rate proportional to a number of partnerships in the population. The full set of partnership links can be thought of as forming a network, through which contact processes spread. For infection, the rate is 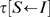, where the term in brackets is the number of susceptible-infectious pairs in the population and 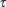 is the transmission rate constant, and for tracing, the rate is 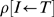, where the term in brackets is the number of infectious-traced pairs in the population and 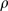 is the tracing rate constant. We have introduced here a notation in which a arrow is drawn from a state that transmits across the link to the state that is affected by the transmission, which will become important when we consider triples in addition to pairs.

To consider the impact of network structure, in particular clustering, on the efficacy of contact tracing, we consider a scenario in which an infection with underlying *SIS* dynamics is at its endemic equilibrium, and then contact tracing is introduced and the numbers infectious measured over time. This requires a dynamical model, and so we now turn to two complementary methods that we use to study the system in question: ODE-based models and stochastic simulation.

### ODE-based models

Models based on ordinary differential equations (ODEs) are widely used in infectious disease modelling. We present here a series of ODE systems that can be used in the context of network models, starting with mean-field approaches, and moving on to pairwise models. We have found that, for application to contact tracing, mean-field models and existing pairwise closures are inadequate and so we develop an improved pairwise model to study this system.

#### Mean-field models

For *SIS* dynamics with transmission rate 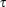 across a network link and treatment rate 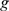 on a large network, the expected numbers of susceptible and infectious individuals evolve according to the following exact, but unclosed, set of equations.
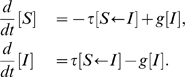
(1)In our notation, 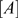 refers to the number of individuals in state 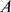, 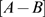 and 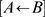 to the number of pairs with one individual in state 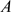 and one in state 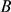, and a directed arrow on the right hand side of a differential equation denotes the direction of transmission for a contact process.

To produce a mean-field model, we use a low-level closure that approximates pairs in terms of individuals.
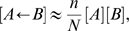
(2)where 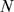 is the number of nodes in the network, and 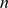 is the number of links per node. For *SIR* dynamics, improvements of this scheme are possible that have a factor of 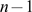 in the numerator of (2) in the place of 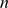, representing the fact that after the first infection, each infected individual in an unclustered network will have one fewer link due to the individual they were infected by. For clustered networks, *SIS* dynamics and contact tracing, all of which we are considering here, it is not clear that a similar argument can be used and so we keep the factor of 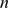.

#### Pairwise models

In pairwise models, rather than using assumptions like (2), equations for the pair-level variables that appear on the right-hand side of (1) are written down, leading to triple-level variables that are then closed in terms of pairs.

The starting point for our analysis is the standard pairwise model for *SIS* dynamics [18], with transmission rate 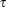 across a network link and treatment rate 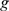. This consists of the unclosed equations (1) above, together with the following equations for pairs.
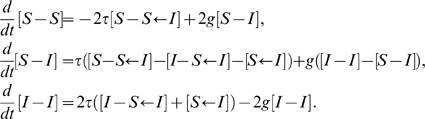
(3)Here, 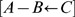 is the number of nodes of type 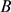 connected to both an 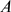 and a 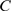, which may or may not be connected themselves. We have continued use of the notation in which a directed arrow on the right hand side of a differential equation denotes the direction of transmission for a contact process, as explained above.

The equations (3) are, like (1), exact, but to produce an integrable system it is necessary to introduce a system of spatial closure. The standard approximation for a population of size 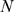, with exactly 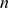 links per node and a clustering coefficient of 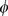
—defined as the ratio of triangles to triples in the network—was derived in [19] and is:
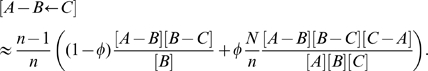
(4)For clarity about the definition of 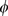, where the network adjacency matrix is 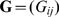, then
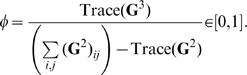
(5)


There are two problems with the approximation (4) that are particularly relevant for the question of contact tracing. The first of these is that we would like to preserve the pair-level relation 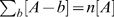. For the dynamical system (3), this pair-level relation will be preserved over time provided the following triple-level equation holds:
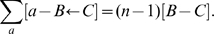
(6)Equation (6) above holds for the standard closure for unclustered networks, but fails to be satisfied for non-zero clustering. The second problem with the standard closure is the question of how triangles of three infected individuals behave during the early asymptotic stage of an epidemic, where all dynamical variables are governed by the proportion of the population that is infectious, 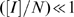. While for pure *SIS* dynamics these triples are not dynamically important, when we come to consider contact tracing similar terms will become relevant. Under (4) and assuming the prevalence of infection is very low, the proportion of unclosed triples composed of three infected individuals is proportional to 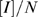 as expected. However, under (4), the proportion of triangles where all three individuals are infected is not small (and does not scale with 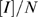); clearly, this is inconsistent and should be rectified in any improved closure.

Motivated by these two considerations, we propose an alternative that respects (6) and has appropriate polynomial dependence on 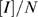 during the early epidemic.
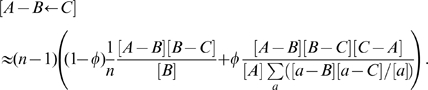
(7)This closure breaks the standard symmetry between 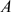 and 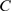, however if contact processes are consistently identified on the right hand side of ODE systems like (3) using arrows, then this is not conceptually problematic.

For the rest of this paper, we call pairwise models based on the closure (7) improved pairwise models; while pairwise models based on (4) are called standard pairwise models.

#### Full pairwise system

Putting together all four processes for our model with tracing, our pairwise system consists of the following exact equations together with the closure approximation (7).
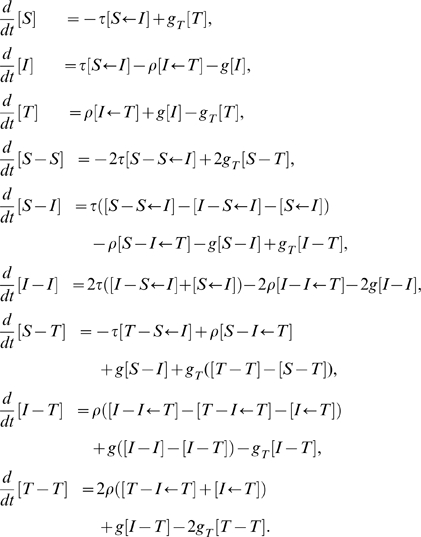
(8)We will also consider, for comparison, these equations closed using (4), and mean-field models.

### Simulation

A complementary approach to pairwise models comes from individual-based, stochastic simulation where an explicit network is generated and dynamical processes are simulated using Monte Carlo methods.

In order to provide a good comparison between pairwise models and simulation, we generate explicit networks that are designed to introduce structure to the population along the lines that we have been considering, by introducing finite neighbourhood size and clustering, without introducing other significant structural features. This enables us to test results derived using pairwise equations against stochastic results. It also complements our general approach of looking at the implications of finite neighbourhood size and clustering *ceteris paribus*, as an aid to intuitive understanding of the impact of population structure on disease and intervention dynamics.

While other methods exist to generate networks with significant clustering coefficients, such as [20]–[22], and some special clustered networks have the attractive property of begin easily generated and analysed [23],[24], we use simple rewiring methods that are easily described and whose implications for global network structure can be readily understood, but which limit us to a smaller region of network parameter space. Most importantly, we find that giant component sizes for networks generated using our methods typically exceed 99%.

#### Creation of a homogeneous random network

In order to create a homogeneous random network, we firstly generate a one-dimensional ring with 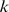-th nearest neighbour links. Since we consider networks where 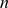 is even, we set 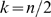, and then make five cycles through every node 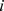, and for each of that node's links 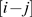, swap with a random link 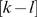 as below,
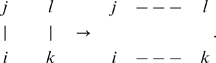
(9)This generates a homogeneous random network free from dynamically relevant biases.

#### Increasing clustering

In order to increase the clustering coefficient for a network, whilst keeping degree distribution constant, we use a new rewiring method that we call the ‘big V’. This means making the following network re-wiring for a ‘V’ of nodes 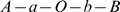 as below,
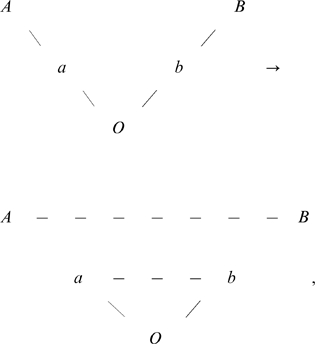
(10)provided the rewiring does not reduce the clustering coefficient overall. Clearly, such a rewiring does not modify the link distribution, but does increase the clustering coefficient. Empirically, we find that at low neighbourhood sizes, this method generates networks with clustering parameters up to 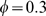 before running out of possible rewirings. Whether alternative methods could yield larger values of 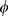 without either a significant reduction in the giant component size or other dynamically relevant biases remains an interesting question, however the levels of clustering given by this rewiring are sufficient to demonstrate the qualitative epidemiological effects in which we are interested. Other recent work making use of this rewiring includes [25],[26].

#### Stochastic dynamics

We simulate *SIS* dynamics with tracing on a network using a standard continuous-time algorithm [27]. The implementation of such algorithms, and the differences between them and discrete-time equivalents, in the context of epidemic models is discussed in [28, Chapter 6]. Since the two contact processes involved (infection and tracing) both involve the explicit network, our model is essentially individual based.

### Parameterisation

For our baseline network parameters, we set 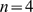 to determine the effects of finite neighbourhood size and clustering. We also take the network size to be 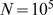 to produce little variability due to stochastic effects after the initial stages of an epidemic. Our main aim is to measure the effects of clustering, 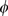, and this is varied between 0 and 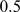. The recovery rate, 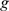, can be formally set to 1 through non-dimensionalisation, and we set 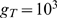 to achieve separation of timescales. Our epidemiological motivation for this separation is the expected difference in the time from infection to detection and treatment, and the time taken to notify sexual partners. For emerging respiratory infections, such a separation of timescales would, of course, not exist.

The other dynamical rates, 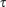 and 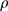 are fixed indirectly. For the tracing rate, 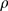, we vary the proportion of contacts successfully traced, 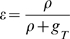, between 0 and 1, which then determines 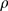. For the infection transmission rate, 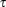, we need methods for fitting to a given endemic equilibrium, in both stochastic and ODE contexts.

#### Pairwise transmission fitting

In the case of fitting to an endemic state, we solve the algebraic equations generated by setting
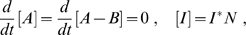
(11)in equations (8), giving a transmission rate 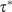 that yields the default endemic equilibrium, 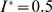.

#### Stochastic transmission fitting

For computational efficiency, we use the following method to find the transmission rate 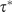 needed to sustain a given endemic prevalence 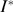 at constant treatment rate 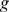:

1. Each individual is set as infectious with probability 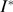 (and conversely, the probability of being set susceptible is 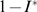).
2. A random 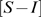 pair is chosen, and the susceptible individual is infected.
3. A random infectious individual is placed into the susceptible class.
4. Steps 2 and 3 are repeated until spatial structure is equilibrated, and then averages 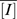 and 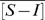 of the number of infectious individuals and susceptible-infectious pairs are taken over a further set of iterations of 2 and 3.
5. The transmission rate is then given by 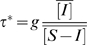.

While this method is not simply described, it is accurate and, most importantly, computationally efficient.

## Results

### Dynamics in the absence of tracing

**Figure 2:**
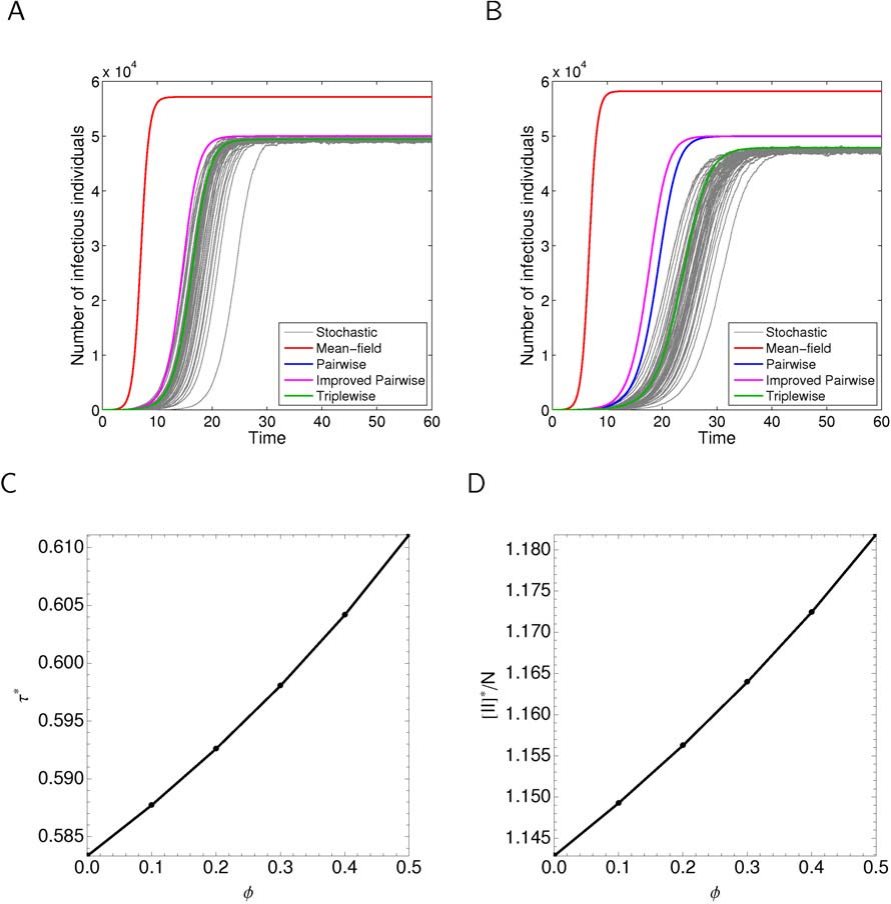
Features of *SIS* dynamics where the transmission rate 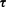 is set in the improved pairwise model to give constant endemic state 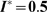. Other parameters are set at their default values: 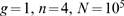. The clustering coefficient 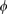 is set at either A: 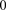 or B: 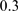, and different ODE approaches are compared to simulation. Also shown are the values of C: 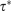, the transmission rate, and D: 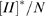, the relative weight of infectious-infectious pairs, at the endemic state as 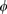 is varied while holding 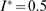.

Figure 2 shows the comparison of stochastic simulation on networks of the type we have been considering with both mean-field *SIS*, standard pairwise, improved pairwise, and also the triplewise model of [29]. This demonstrates good agreement between simulation and network ODE models, but poor agreement with the mean-field model. The inclusion of the triplewise model shows that disagreements between pairwise models and simulation in the clustered network are largely due to higher order structure, however these effects are nowhere near as large as the differences between mean-field and pairwise models. Since triplewise models involve a massive increase in computational burden, we do not consider that in this case their use is justified.

The results of Figure 2 were obtained by fitting the improved pairwise model to a given endemic state, 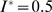. The impact of this fitting on the transmission rate and number of 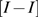 pairs, while varying the clustering coefficient 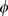, is shown in Figure 2, panes C and D.

### Impact of network structure on contact tracing

**Figure 3:**
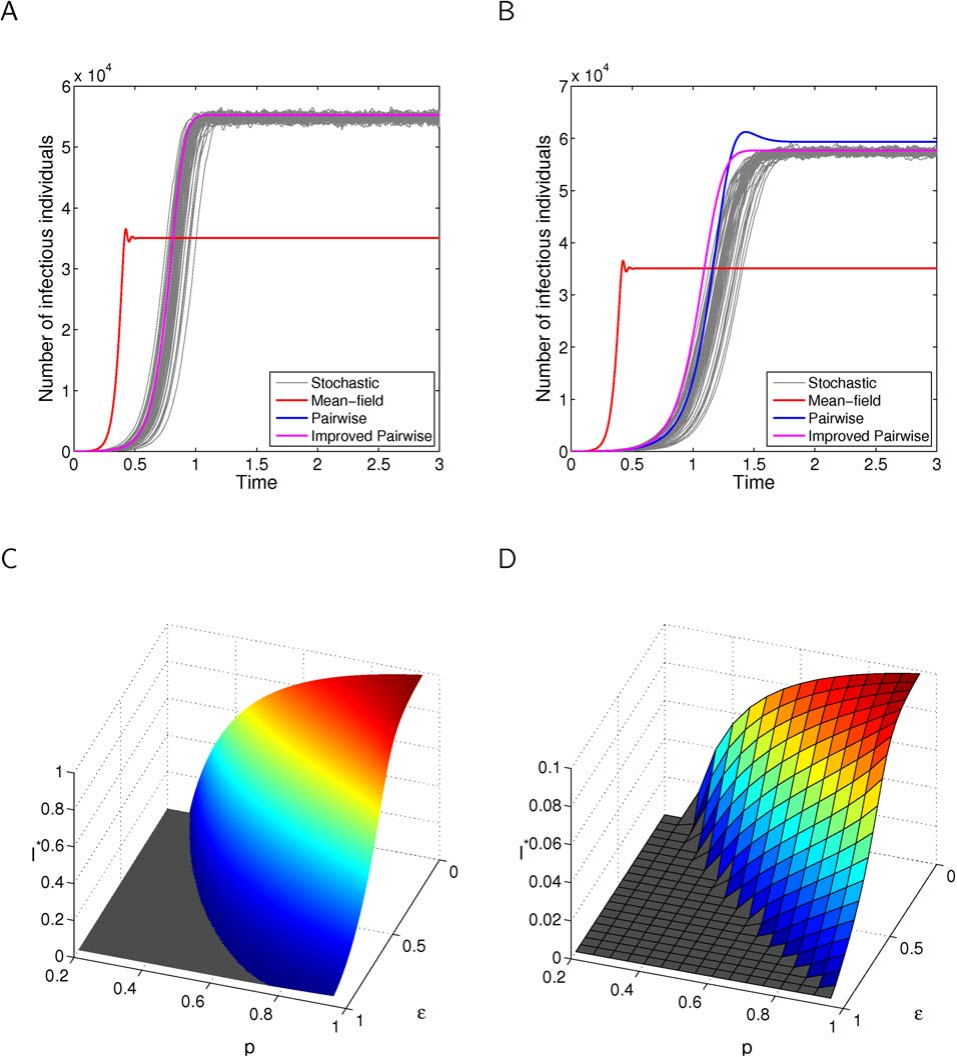
Comparison of infection curves for *SIS* dynamics with contact tracing. Clustering, 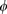 is set to either A: 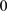 or B: 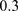, with contact tracing success, 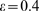 and infection rate, 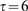. Other parameters are set to their default values: 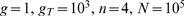. The best agreement is between simulation and the improved pairwise model, with the mean-field approach qualitatively wrong. Sweeping over 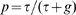 and 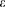 for 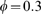, the endemic states predicted by C: the improved pairwise model, and D: stochastic simulation on 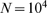 nodes, are in good agreement except where prevalences are low.

The need to incorporate network structure into models that involve contact tracing is shown by Figure 3. Panes A and B show predictions of prevalence over time for several models, which demonstrate that while both pairwise approaches are in good agreement with simulation, the failure of the mean-field model is dramatic—and similarly large failures can be observed in several other regions of parameter space.

For the case of a clustered network in Pane B, the agreement between pairwise models and simulation becomes slightly worse than for the unclustered network results of Pane A, with the improved pairwise model providing a closer fit. Most importantly, the improved pairwise model is in qualitative disagreement with simulation—while both mean-field and standard pairwise models predict a peak in infection before reaching the endemic state, which is not seen in either the improved pairwise model or simulation. We therefore use the results of Panes A and B to rule out the use of mean-field and standard pairwise models. This leaves the improved pairwise model, which we systematically compare to simulation in Panes C and D. Since both the improved pairwise model and simulation depend on underlying parameters in the same way, they form a complementary pair of approaches to the study of contact tracing in clustered populations. The only exception to this is the case of low prevalence of infection, where stochastic effects become important and the stochastic model predicts extinction at higher transmission rates than the pairwise model.

### General results

**Figure 4:**
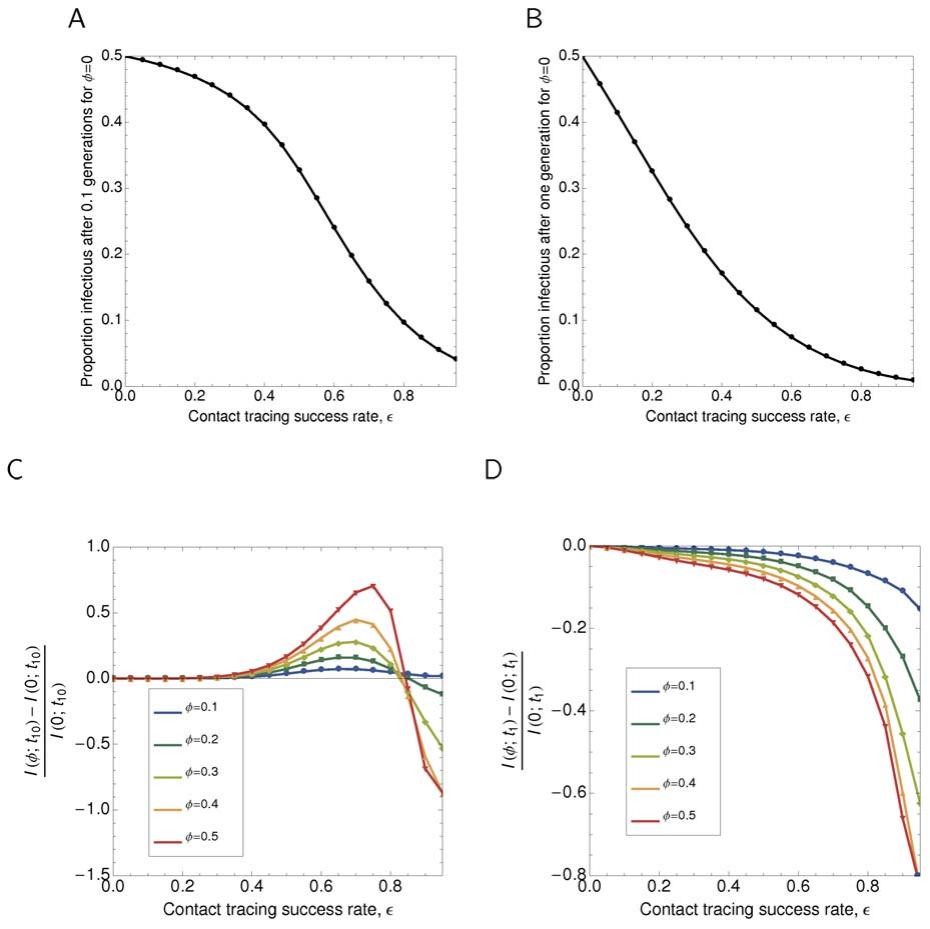
Impact of clustering on efficacy of contact tracing away from the equilibrium state with half of the population infectious. The system is started in the endemic equilibrium, and then for A, B: 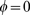 or C, D: 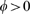, the level of infection is measured at times A, C: 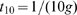 and B, D: 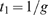. Results are obtained in the improved pairwise model, with contact tracing success 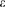 varied between 0 and 1. Other parameters set to defaults: 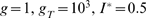.

We consider the effects of clustering on the efficacy of contact tracing using pairwise models by starting the system at the endemic state in the absence of any contact tracing. We then introduce tracing at a success probability 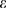, and allow the system to evolve away from the endemic state for 0.1 and one generations (time periods 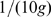 and 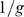 respectively, corresponding to policy evaluation after a number of months and a number of years for an endemic STI) and measure the numbers infectious. This gives the results in Figure 4, which show that clustering increases the efficacy of contact tracing at a given success rate at one infectious generation, but not at 0.1 generations, depending on the actual tracing success rate. Pane C shows this variety of responses, where clustering is more effective for large success rates at this small time—the very large rates require still smaller times to demonstrate this effect, since after 0.1 disease generations they have passed into the regime where clustering leads to less effective tracing.

**Figure 5:**
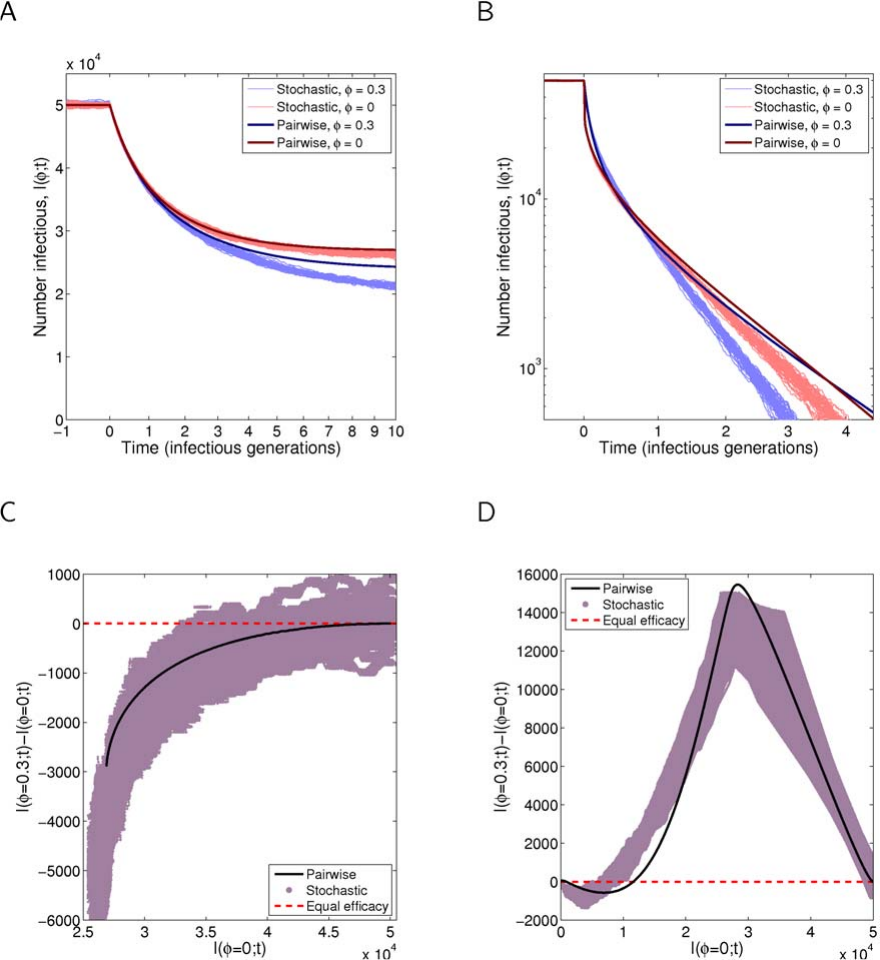
Temporal and parametric behaviour of contact tracing. The system is started in the endemic equilibrium, and then contact tracing is started with success rate 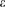 equal to A, C: 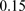 or B, D: 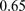. Other parameters are at default values: 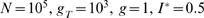. The level of infection over time for different values of clustering 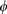 are shown in Panes A and B, while in C and D the difference between clustered and unclustered is shown parametrically against the unclustered result—above the dotted line, clustering implies more efficacious tracing, and vice versa.

The results shown in Panes C and D of Figure 2 provide a guide to intuition to explain these results. Clustering increases the number of 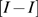 pairs present at a given endemic state, and contact tracing can be viewed as hyper-parasitism on the network of infected individuals. This means that clustering can be expected to enhance the efficacy of contact tracing by increasing the neighbourhood size of the infected sub-network. On the other hand, to explain a constant level of endemic infection as clustering is increased, a larger underlying rate of transmission must be present, which will undermine tracing as an individual left untouched by a wave of tracing will reinfect their immediate neighbourhood more quickly. Exactly which parameter choices allow either effect to dominate is not clear, except that lower levels of tracing success always cause clustering to increase the efficacy of tracing. Otherwise, it appears that the impact of clustering on contact tracing needs to be evaluated on a case-by-case basis.

### Individual trajectories

To see the dynamics of infection that generate the results in Figure 4, we plot stochastic and improved pairwise temporal and parametric dynamics for two exemplar values of contact tracing success, 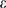, in Figure 5. For 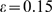, we see in Pane A that the system settles over time to a different endemic state, and in Pane C that this involves consistently lower levels of infection in the clustered system than the unclustered system, meaning that clustering has enhanced the efficacy of contact tracing.

By contrast, for 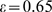, we see in Pane B that contact tracing drives infection to extinction, and from Pane D that this involves firstly higher levels of infection in the clustered system and then lower levels of infection for both the pairwise and stochastic models. We see a final reversal of the impact of clustering in Pane B, which is present in only the pairwise system: at longer times clustering again reduces the efficacy of contact tracing. At this point, stochastic variability in simulations has become highly significant and so we would not expect the two models to agree, since the pairwise equations should only hold in the limit where stochastic effects are negligible. To simulate in this regime would require extremely large population sizes, perhaps beyond what would ever be considered for realistic human scenarios.

## Discussion

We have provided an intuitive and general framework in which to study the impact of network clustering on the efficacy of contact tracing in the control of infectious disease. This has produced three major results.

Firstly, the effects of contact tracing often cannot be accurately captured by mean-field models, necessitating a modelling approach that incorporates network structure.

Secondly, we have demonstrated that due to the increased number of infectious-infectious pairs seen in clustered networks at a given pathogen burden, contact tracing at a fixed, relatively low success rate will be more effective at larger levels of clustering than at the same fixed success rate without clustering.

Finally, we have demonstrated that this increased efficacy is not completely general, and is reversed for large tracing success rates at certain times. This demonstrates the need to be cautious in the consideration of the epidemiological effects of a network property as subtle as clustering—unfortunately it is not possible to obtain a general ‘rule of thumb’ for its impact.

Our approach has been to consider the impact of clustering on a network with fixed, finite neighbourhood size, in the absence of other known important dynamical effects such as risk structure and assortativity. The complexity of even our simplified problem provides justification for our approach, however it would be of significant interest to see how these quantities interact with each other. The full impact of higher order structure than triangles is also, as suggested by our stochastic results, likely to be important.

Another important difference may manifest itself if we were to consider a disease with long-lasting immunity, obeying *SIR* dynamics, or other compartmental structure, including complex intervention strategies and comparable tracing and recovery timescales. Our preliminary work in this direction suggests that our novel result about clustering reducing contact tracing efficacy can be extremely significant in other contexts, however a full consideration of this would take us significantly beyond the aims of the present work.
